# Notch signaling negatively regulates BMP9-induced osteogenic differentiation of mesenchymal progenitor cells by inhibiting JunB expression

**DOI:** 10.18632/oncotarget.22763

**Published:** 2017-11-30

**Authors:** Nan Wang, Wei Liu, Tao Tan, Chao-Qun Dong, Duan-Yang Lin, Jun Zhao, Chang Yu, Xiao-Ji Luo

**Affiliations:** ^1^ Department of Orthopedics, The First Affiliated Hospital of Chongqing Medical University, Chongqing 400016, P.R. China; ^2^ Department of Neurosurgery, The First Affiliated Hospital of Chongqing Medical University, Chongqing 400016, P.R. China; ^3^ Department of Orthopedics, The Affiliated Children’s Hospital of Chongqing Medical University, Chongqing 400014, P.R. China

**Keywords:** BMP9, Notch signaling, mesenchymal stem cells, osteogenic differentiation, JunB

## Abstract

Although interaction between BMP and Notch signaling has been demonstrated to be crucial for osteogenic differentiation of mesenchymal stem cells (MSCs), the precise molecular mechanism remains unknown. Here, we show that Notch intracellular domain (NICD) overexpression inhibits BMP9-induced C3H10T1/2 cell osteogenesis *in vivo* and *in vitro*. Our results show that activated Notch signaling results in down-regulation of Runx2 and early osteogenesis differentiation factors, without affecting p-Smad1/5/8 expression, and that blocking Notch signaling with DAPT (N-[N-(3,5-difluorophenacetyl)-L-alanyl]-S-phenylglycine t-butyl ester) significantly increases p-Smad1/5/8 expression. Interestingly, Notch signaling also regulates the cell cycle by increasing PCNA (proliferation cell nuclear antigen) and CyclinD1 expression. Furthermore, similar results were obtained by ectopic bone formation and histological analyses, indicating that Notch signaling activation significantly inhibits BMP9-induced MSC osteogenic, cartilage and adipogenic differentiation. Moreover, we are the first to show that Notch regulates by suppressing JunB synthesis and that the negative effect of Notch is partially reversed by treatment with the JunB activator TPA (12-O-tetradeca-noylphorbol-13-acetate). Our findings demonstrate that Notch signaling significantly enhances cell proliferation but inhibits MSC osteogenic differentiation induced by BMP9 via JunB protein suppression rather than by BMP/Smad signaling regulation.

## INTRODUCTION

Bone defects and non-union are major problems in orthopedic clinical work. Tissue engineering technology for clinical repair of damaged bones has wide application prospects, and mesenchymal stem cells (MSCs) are intensely researched in this regard [1, 2]. Indeed, because of their capacity to differentiate into multiple cell lines and their wide variety of sources, MSCs have great potential for healing injured tissue [3-6]. Therefore, it is necessary to define the specific mechanism underlying the key pathways as well as the interactions between different signaling pathways that regulate MSC differentiation into osteoblasts. Previous studies have shown that various pathways, such as BMP, Wnt, Notch, and Hedgehog, are involved in regulating MSC osteogenic differentiation [7-11], and knowledge of such factors is necessary for developing druggable targets to promote bone formation.

*Bone morphogenetic proteins* (BMPs) have been demonstrated to induce osteogenic MSC differentiation both *in vitro* and *in vivo.* Among the BMP family, a previous study showed that BMP9 is the strongest induction factor for MSC osteogenesis [12]. Although interfering with BMP signaling impairs chondrogenic or osteogenic differentiation and induces skeletal patterning defects [13, 14], the mechanism by which factors that accelerate BMPs induce MSC differentiation toward osteoblastic cells remains unclear. BMP-induced osteogenic differentiation is a multi-factor process that involves Notch participation. Because it translates cell-cell interactions into specific transcriptional programs, evolutionarily conserved Notch signaling has a crucial function in cell fate determination and various developmental processes [15, 16]. In mammals, the canonical Notch pathway is activated when Jagged 1 or 2 or Delta-like 1, 3, or 4 Notch ligands bind to cell-surface Notch receptors (Notch1, Notch2, Notch3, or Notch4) on neighboring cells. The receptors then undergo a series of proteolytic cleavages, ultimately resulting in the release of the Notch intracellular domain (NICD), and NICD then translocates to the nucleus to activate gene expression of target genes such as Hes and Hey via formation of a transcriptional complex with recombination signal binding protein for immunoglobulin kappa J region (RBP-Jk) and Mastermind-like proteins [17, 18]. The literature to date suggests that the role of Notch signaling in osteogenesis differentiation is contradictory but that it is crucially involved in regulating BMP-induced osteogenic differentiation of MSCs [10, 11, 19].

Overall, cross-talk between the BMP and Notch pathways has not been clarified in MSC osteogenic differentiation. Moreover, the complexities of pathway interactions have led to some seemingly contradictory reports, creating an often confusing and disjointed knowledge base [11, 20]. On the one hand, researches has indicated that the negative effect of Notch on BMP-induced osteogenic MSC differentiation might occur through NICD, Hey1 or Hes1, which inhibit the activity of Runx2 via direct binding [19-21], or by decreasing expression of Smad proteins and their target genes [22, 23]. In addition, deletion of RBP-Jk in MSCs enhances osteogenic activity through up-regulation of BMP signaling by relieving Smad1/5/8 complex inhibition [24]. On the other hand, it has been reported that Hey1 or Hes1 can synergistically enhance BMP-induced MSC osteogenic differentiation [20, 25, 26]. In general, the majority of studies have simply illustrated that Notch signaling activation can down-regulate Runx2 activity, yet the specific mechanism underlying this interaction has not been elucidated. One study found that BMP-Smad signaling does not directly induce Runx2 expression and that an additional step involving de novo protein synthesis is required for BMP-Smad-induced synthesis of JunB, which functions as an upstream activator of Runx2 expression [27]. Another report revealed that JunB knockout results in severe osteopenia [28].

Thus, for clear mechanistic insight into how the Notch pathway regulates BMP signaling in osteogenic differentiation of MSCs, we investigated potential cross-talk between these two cascades. We found that activation of Notch signaling can inhibit BMP9-induced MSC osteogenesis by suppressing Runx2 expression. Further experiments revealed that this inhibitory effect was not mediated through down-regulation of total Smad1/5/8 expression or Smad1/5/8 phosphorylation but via action against another target, JunB, to suppress Runx2 expression.

## RESULTS

### Notch activation inhibits BMP9-induced MSC osteogenic differentiation

**Figure 1:**
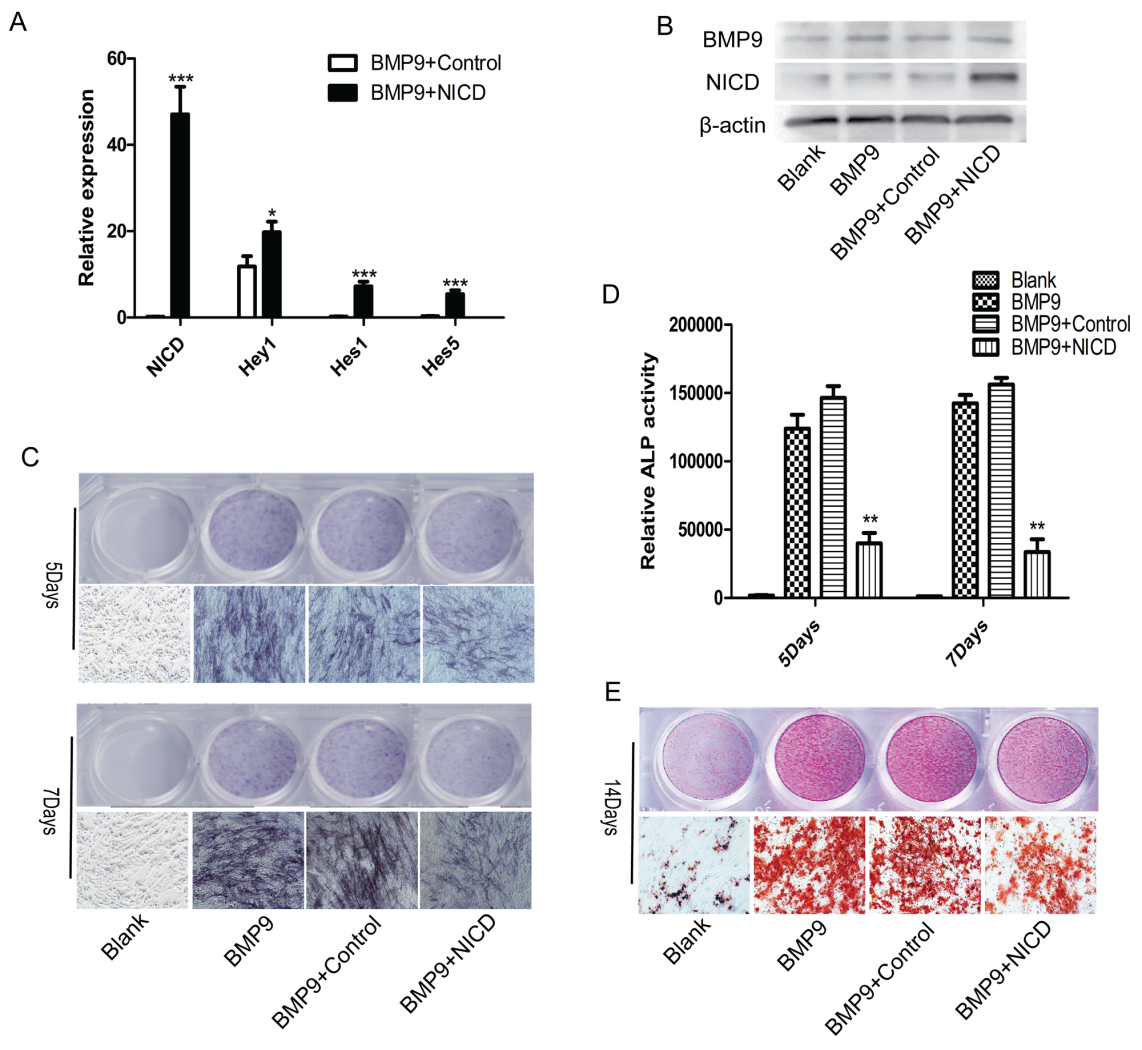
Notch activation inhibits BMP9-induced C3H10T1/2 osteogenic differentiation **(A)**, **(B)** The transfection efficiency of the NICD plasmid was detected by expression of NICD or the Notch target gene Hey1, Hes1, Hes5 via RT-qPCR and western blotting. **(C)**, **(D)** Representative images of ALP staining. ALP staining and activity were detected at 5 and 7 days after cells were transfected with the NICD plasmid. **(E)** Representative images of Alizarin red staining. Formation of calcium nodules was detected after cells were treated with the NICD plasmid or Ad-BMP9 for 14 days. RT-qPCR data represent the means ± SD of three independent experiments performed in duplicate; the control gene expression level was set at 1. The data were normally distributed, and they were analyzed using one-way ANOVA (n=3). ^*^P< 0.05, ^**^P< 0.01 vs the control at the same time point.

Previous studies have often used NICD to activate Notch signaling [32, 33], and we also employed an NICD overexpression plasmid in the current study. At 72 h post-transfection, RT-qPCR analysis revealed increased expression of NICD, Hey1, Hes1 and Hes5 by 47-fold, 1.7-fold, 7.2-fold and 5.4-flod respectively (Figure 1A). In addition, BMP9 and NICD expression at the protein level were also significantly enhanced, consistent with the level of RNA expression (Figure 1B). To determine the effect of Notch signaling on BMP9-induced MSC osteogenesis, cells were transfected with Ad-BMP9 or an plasmid NICD and further cultured. The results showed that BMP9-induced formation of ALP was dramatically inhibited in the NICD-treated group, and this inhibitory effect was further confirmed by ALP activity measurements, which reflect the absolute expression level of ALP (Figure 1C, 1D). Moreover, Alizarin red staining showed dramatically decreased formation of calcium salt nodules in the NICD-treated group (Figure 1E). Taken together, these data demonstrate that activation of Notch signaling may have a negative effect on the regulation of MSC osteogenic differentiation.

### Notch signaling activation inhibits BMP9-induced MSC osteogenic differentiation through down-regulation of Runx2 expression but does not influence Smad1/5/8 activity

**Figure 2:**
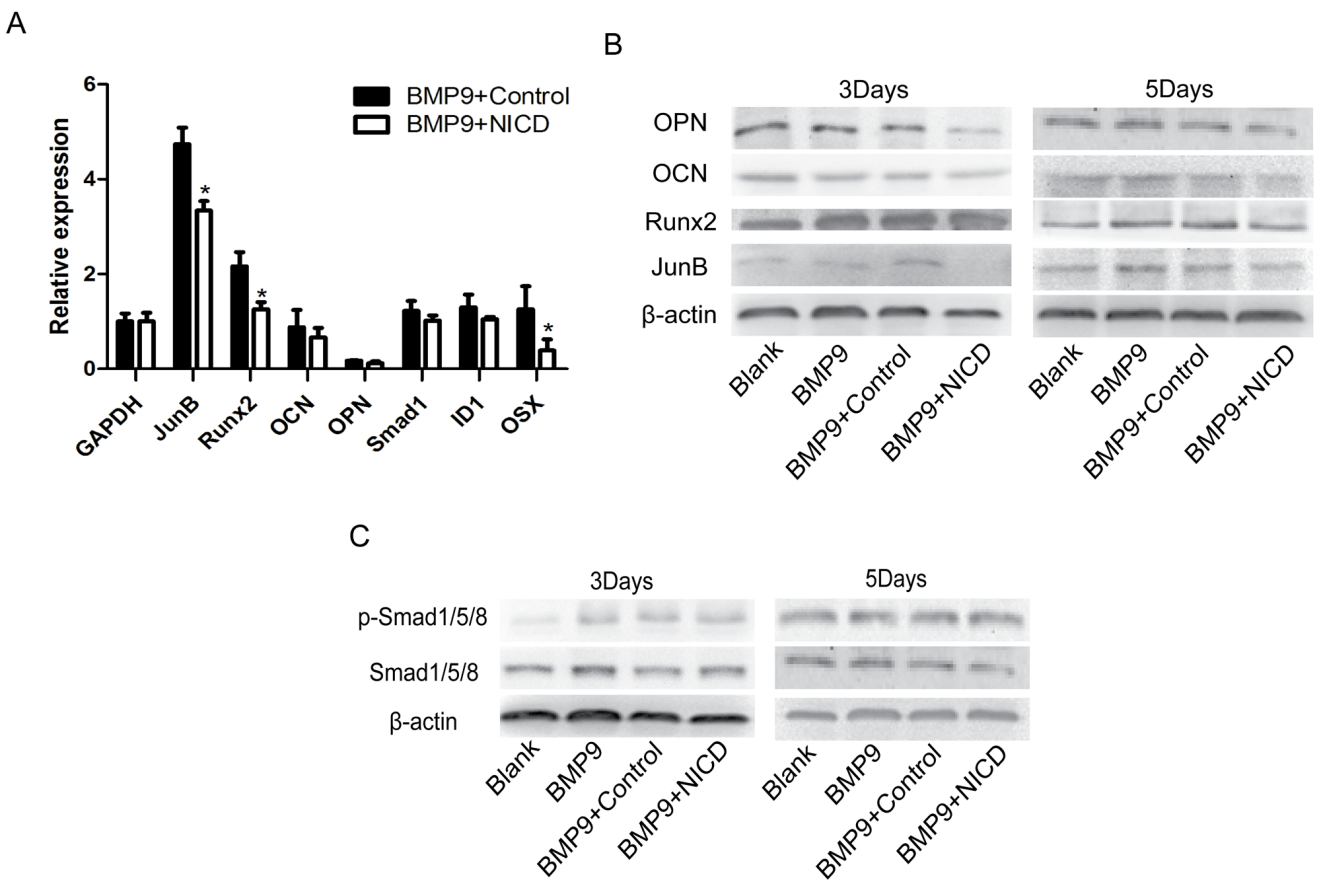
Notch activation inhibits BMP9-induced osteogenic differentiation through down-regulation of Runx2 expression NICD plasmid-treated cells were at the indicated time points harvested for total RNA and protein isolation. **(A)** Expression of osteogenesis related genes Runx2, OCN, OPN, and OSX was detected by RT-PCR after cells were treated with the NICD plasmid or Ad-BMP9 for 48 h. **(B)** Western blot analysis of OCN, Runx2, and OPN expression at the indicated time points. **(C)** Total SMAD 1/5/8 and p-SMAD 1/5/8 expression was analyzed in each treatment group by western blotting. RT-qPCR data represent the means ± SD of three independent experiments performed in duplicate; the expression level of the control gene GAPDH was set at 1. The data were normally distributed, and they were analyzed using one-way ANOVA (n=3). ^*^P< 0.05, ^**^P< 0.01 vs the control at the same time point.

Next, we performed experiments to delineate the underlying mechanism of the observed inhibited osteogenic ability in the NICD group. First, expression of osteogenesis-related genes Runx2 and OSX was found to be significantly inhibited by NICD (Figure 2A). Interestingly, lower expression levels of the late osteogenic markers osteopontin (OPN) and osteocalcin (OCN) compared to basal expression at the early stage of BMP9-induced differentiation were found (Figure 2A). Furthermore, Runx2, OPN and OCN expression was investigated by western blot analysis, and the results showed a trend similar to that observed by RT-qPCR (Figure 2B). Unexpectedly, NICD did not have any significant effect on SMAD 1/5/8 or p-SMAD 1/5/8 levels (Figure 2C). Furthermore, JunB was inhibited not only at the RNA level but also at the protein level (Figure 2A, 2B). These results suggest direct regulation between the Notch and BMP pathways, with JunB possibly being the convergence point, which has not been reported previously.

### JunB may be a new target through which activation of Notch signaling inhibits BMP-Smad signaling-induced MSC osteogenic differentiation

**Figure 3:**
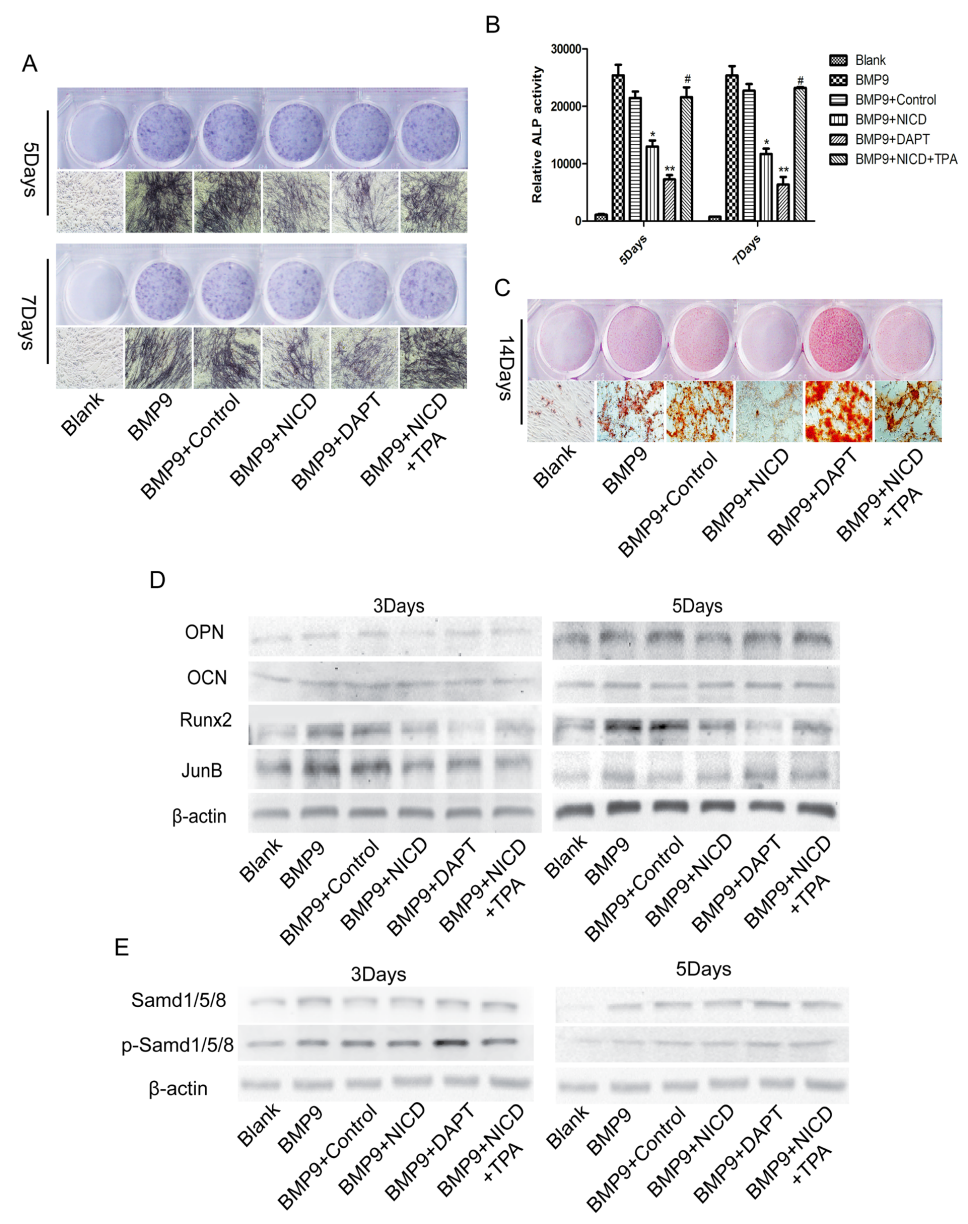
JunB is involved in Notch-induced low Runx2 expression in association with BMP9-induced MSC osteogenic differentiation **(A)**, **(B)**, **(C)** Representative images of ALP and Alizarin red staining. The effects of NICD on ALP staining and activity and calcium nodule formation were detected at the indicated time points after cells were treated with TPA (100 ng/ml). **(D)** Protein expression of Runx2 and other related proteins was detected in NICD plasmid-treated cells after treatment with TPA (100 ng/ml). **(E)** Western blot analysis of total SMAD 1/5/8 and p-SMAD 1/5/8 expression levels in each treatment group. The data were normally distributed, and they were analyzed using one-way ANOVA (n=3). ^*^P< 0.05, ^**^P< 0.01 vs the control; ^#^p<0.05 vs NICD-treated cells.

We then performed experiments to determine the exact effect of JunB on BMP and Notch signaling. In addition, we also assessed BMP9-induced osteogenic differentiation when the Notch signal was completely blocked by DAPT, a γ-secretase inhibitor that blocks all Notch signaling. The negative effect of NICD on ALP expression and calcium nodule formation was dramatically reversed after adding TPA, which is a JunB activator [27, 34] (Figure 3A, 3C), and this result was verified by an ALP activity assay (Figure 3B). Unexpectedly, we found marked inhibition of ALP expression in the DAPT group, whereas calcium deposition was dramatically increased (Figure 3A, 3C). Furthermore, the result confirmed that efficient down-regulation of Runx2 and JunB expression by NICD was notably restored after applying TPA (Figure 3D). Runx2 expression was also significantly inhibited by DAPT but p-SMAD 1/5/8 expression dramatically increased (Figure 3D, 3E). In addition, expression of JunB, OPN and OCN was correspondingly enhanced after applying TPA (Figure 3D). Overall, these results provide a possible mechanism, whereby the suppressive effect of Notch on BMP9-induced osteogenesis is mediated through its influence on JunB.

### Notch signaling is involved in promoting cell proliferation and maintaining bone marrow mesenchymal progenitors

**Figure 4:**
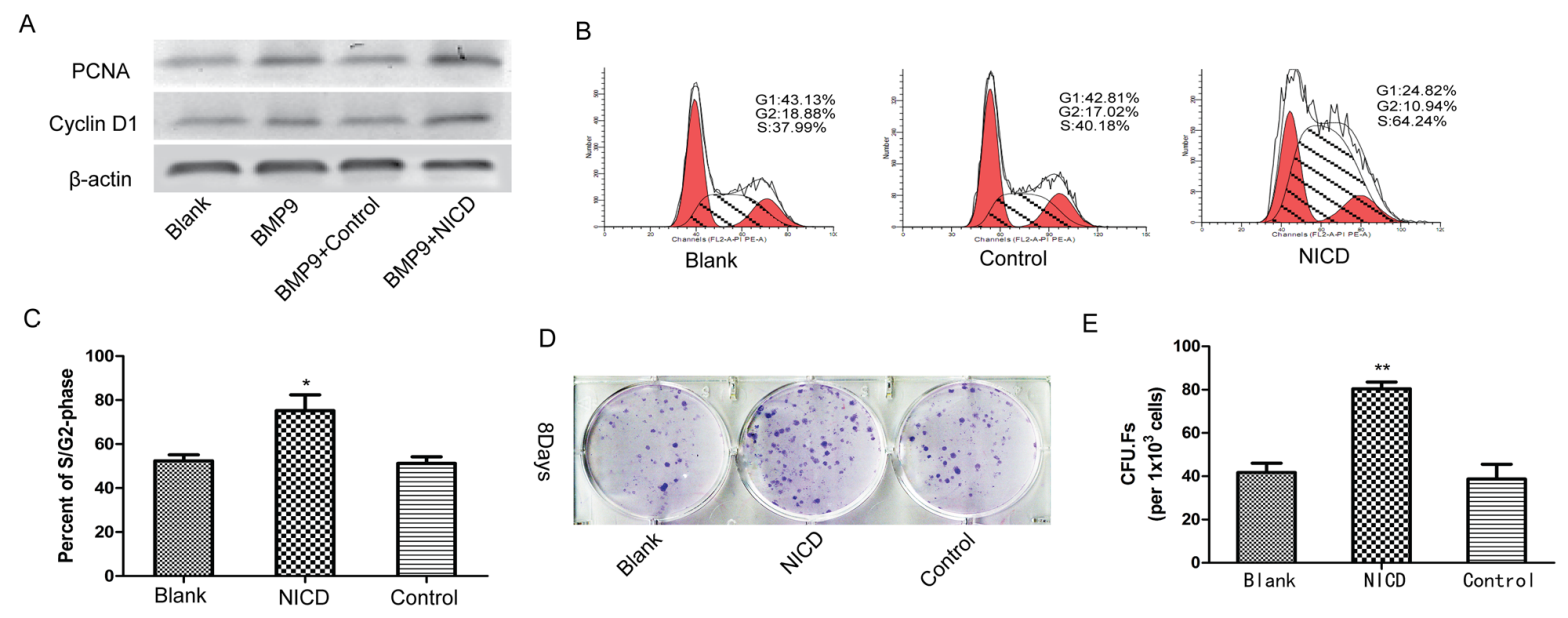
Notch activation is involved in the regulation of MSC proliferation and stemness maintenance **(A)** The cell cycle-related genes PCNA and CyclinD1 were detected by western blot analysis in cells treated with the NICD plasmid for 3 days. **(B)**, **(C)** The cell cycle was detected by flow cytometry after cells were treated with the NICD plasmid for 48 h. **(D)**, **(E)** CFU-F assays were performed to assess the effect of NICD on the colony-forming capacity of cells for 8 days (1×10^3^ cells/dish). Representative images are shown. The data were normally distributed, and they were analyzed using one-way ANOVA (n=3). ^*^P< 0.05, ^**^P< 0.01 vs the control at the same time point.

The association between Notch signaling and the cell cycle has been well documented [35], and studies have reported that the Notch pathway is aberrantly activated in cancer cells [36, 37]; the same types of studies have also been reported in MSCs [19, 21]. Thus, we performed an experiment to determine whether cell cycle regulators are also involved in MSC osteogenesis; the results showed significantly increased PCNA and Cyclin D1 expression in the NICD-treated group (Figure 4A). Flow cytometry results also revealed a significantly increased G2/M cell population after treatment with NICD (Figure 4B, 4C). Moreover, CFU-F assays were used to detected the self-renewal ability of cells, and remarkably, the NICD-treated cells formed significantly more and larger CFU-Fs (Figure 4D, 4E). Hence, these results indicate that Notch signaling activation maintains the stemness of MSCs by suppressing osteogenic differentiation.

### Notch signaling activation inhibits BMP9-induced ectopic bone formation *in vivo*

**Figure 5:**
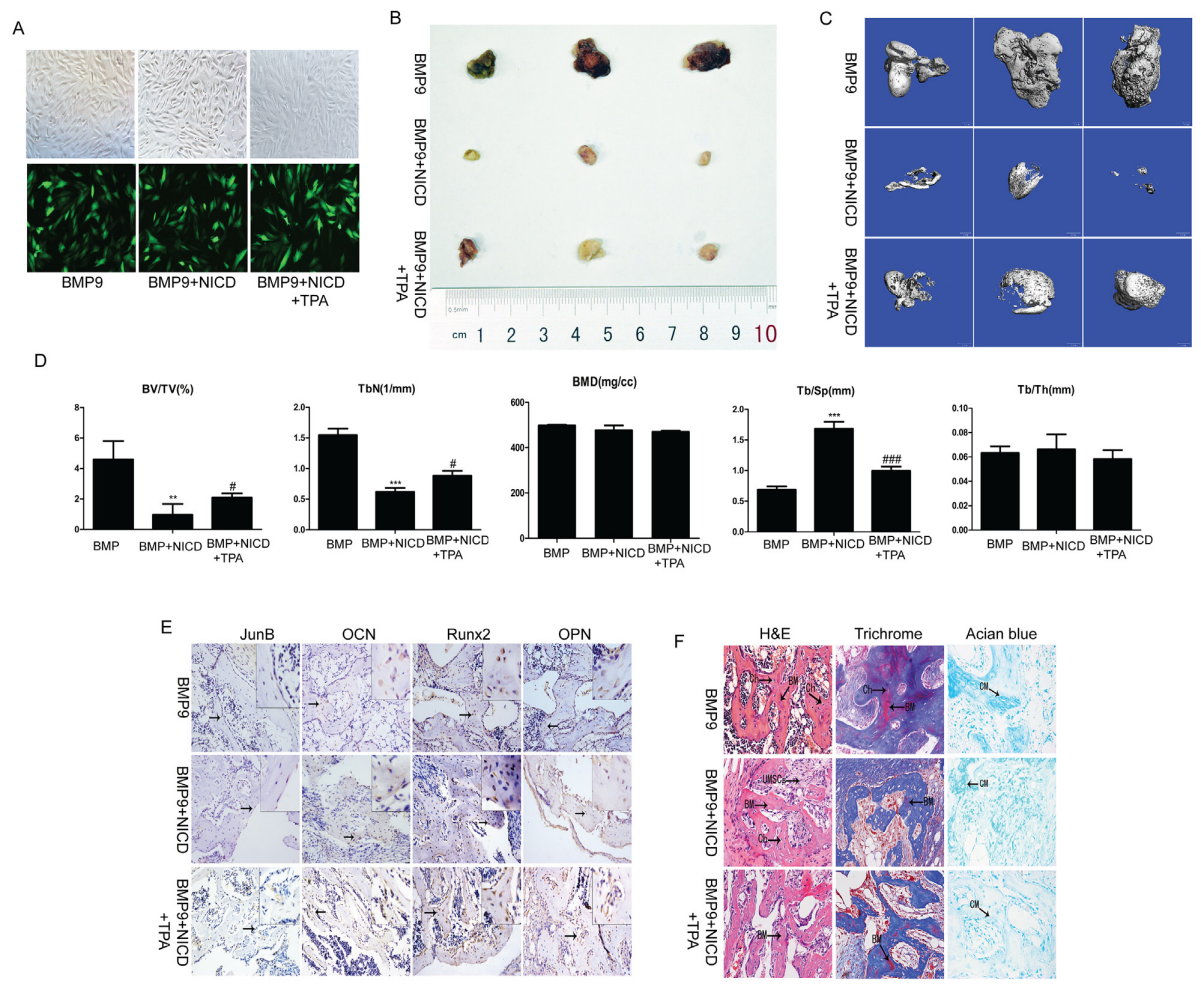
The effect of activated Notch signaling on BMP9-induced ectopic bone formation *in vivo* **(A)** C3H10T1/2 cells were co-transduced with AdBMP-9 or the NICD plasmid. After 24 h, the AdBMP-9 infection efficiency was determined by fluorescence microscopy. **(B)** Macrographic images of ectopic bone mass. **(C)** MicroCT analysis. Retrieved masses were further subjected to microCT scanning. Representative reconstructed 3-dimensional images are shown; the scaling ratio is 1 mm. **(D)** Histomorphometrical analysis of structural bone parameters. Trabecular bone volume (BV/TV; %), trabecular thickness (Tb/Th; mm), trabecular separation (Tb.Sp; μm), and bone mineral density (BMD; mg/cc) were calculated based on microCT scanning data. **(E)** Protein expression of JunB, OCN, Runx2, and OPN was confirmed by immunohistochemical staining in retrieved samples. Representative images are shown. Magnification, ×200. **(F)** Histological staining of retrieved samples. Serial sections of embedded specimens were stained with H&E, Masson’s trichrome and Alcian blue. BM, Bone Matrix; Ch, Chondrocyte; UMPCs, Undifferentiated mesenchymal progenitor cells; CM, Cartilage Matrix. Magnification, ×400.

The above *in vitro* data demonstrate that canonical Notch signaling has an important function in BMP9-induced osteogenic differentiation of MSCs, and we sought to confirm these findings *in vivo* via stem cell implantation experiments. C3H10T1/2 cells were effectively transduced with Ad-BMP9 (Figure 5A); some cells were treated with TPA, followed by further culturing for 24 h. The cells were collected and injected subcutaneously into athymic mice. At 5 weeks, the animals were euthanized, the bony masses were retrieved, and ectopic bone formation was accurately and quantitatively analyzed by microCT. The gross appearance and three-dimensional reconstruction of the retrieved samples indicated that activated Notch signaling inhibited ectopic bony mass formation and that TPA partially reversed the effect of NICD (Figure 5B, 5C). Furthermore, static histomorphometric analysis showed that the bone volume and trabecular number were markedly reduced in the NICD group, whereas trabecular separation was obviously increased. However, no notable difference in bone mineral density or trabecular thickness was observed, similar to the findings that TPA attenuates the effects of NICD (Figure 5D).

The results of immunohistochemistry showed JunB, Runx2, OCN, and OPN expression to be significantly decreased in the NICD-treated group, and as expected, the relative expression of the abovementioned genes was markedly reversed after treatment with TPA (Figure 5E). Furthermore, based on H&E staining, the NICD-treated samples contained fewer ossified matrices and adipocytes, osteoblasts and chondrocytes. Alcian blue staining confirmed a small amount cartilage matrix in the NICD samples, but more bone matrix and osteoblasts and less cartilage matrix was observed in the TPA-treated samples (Figure 5F). Thus, in agreement with our *in vitro* results, these findings support the negative impact of NICD in BMP9-induced osteogenic differentiation of MSCs and that JunB may act as a critical intersection between BMP and Notch pathways.

### Notch signaling activation inhibits BMP9-induced adipogenic differentiation

**Figure 6:**
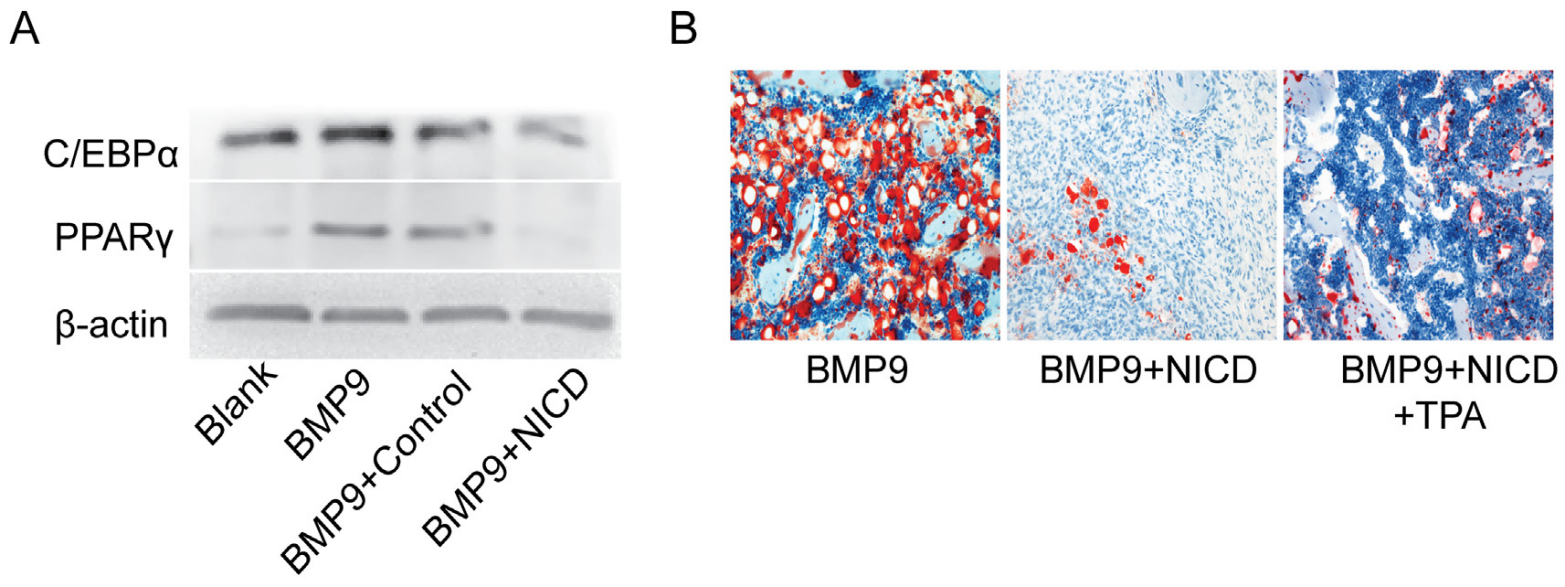
Notch signaling activation inhibits BMP9-induced adipogenic differentiation **(A)** Expression of C/EBPα and PPARγ was detected by western blotting after cells were treated with Ad-BMP9, the NICD plasmid for 9 days. **(B)** Histological staining of retrieved samples. Frozen sections were stained with oil red O. Representative images are shown. Magnification, ×200.

Because osteogenic differentiation is often associated with the adipogenesis process [38], relevant adipogenic indicators were detected *in vitro* and *in vivo*. Western blotting showed that expression of CCAAT-enhancer-binding protein α (C/EBPα) and peroxisome proliferator-activated receptor γ (PPARγ) were markedly inhibited by treating with NICD for 9d when compared with the control group (Figure 6A). Moreover, Oil red O staining powerfully showed that NICD can significantly impair lipid droplet formation when compared with the BMP9 group, and the commitment toward adipocytes was also inhibited in the TPA group (Figure 6B). These findings show that NICD not only suppresses osteogenic differentiation of MSCs but also inhibits the adipogenic process.

## DISCUSSION

Bone regeneration via tissue engineering, which utilizes MSCs grown in an osteoconductive scaffold with osteoinductive growth factors, is a promising approach for bone repair that has been studied for over a decade [39]. BMP2 and BMP7, members of the TGF-β superfamily, are potent osteoinductive growth factors that have been applied in clinical treatment [40, 41]. A previous study showed a significantly stronger ability of BMP9 to induce MSC osteogenesis compared to other TGF-β superfamily subtypes [12, 31, 42]. Multiple signaling pathways are involved in regulating this process [11], yet reports on interactions between the BMP and Notch pathways in MSC osteogenic differentiation regulation are inconsistent, and the specific regulatory mechanism remains unclear [11, 43]. Therefore, we are particularly interested in illuminating the influence of Notch signaling on BMP9 osteoinductive activity.

In this report, we demonstrate that Notch signaling activation due to overexpression of NICD inhibits BMP9-induced MSC osteogenic differentiation, leading to lower ALP expression levels and decreased calcium nodule formation compared to the control group. These results are consistent with previous findings suggesting that Notch singnaling has a negative effect on MSC differentiation [33]. However, opposite trends for early ALP expression, osteogenesis-related gene expression and terminal calcium nodule formation were observed when all Notch signaling was blocked by DAPT. These findings suggest that MSC proliferation may have an important function in the early osteogenic differentiation process but suppresses MSC osteogenic differentiation in the early stage [19, 21, 44] or that different Notch receptor subtypes have different impacts on osteogenesis differentiation [23, 33, 45]. We next investigated the specific regulatory mechanism of Notch activation in BMP9-induced differentiation. Unexpectedly, our results showed expression of Runx2, OCN, and OPN to be suppressed to varying degrees but that the RNA and protein levels of Runx2 were significantly inhibited, indicating that the effect of NICD might be mediated by regulating the expression of factors upstream of Runx2 to influence its output. Furthermore, we examined the direct upstream factor of Runx2, Smad1/5/8; however, both total Smad1/5/8 and p-Smad 1/5/8 levels were not affected by NICD. This result indicates that the inhibitory effect of NICD may impact other unknown factors that regulate MSC osteogenic differentiation.

**Figure 7:**
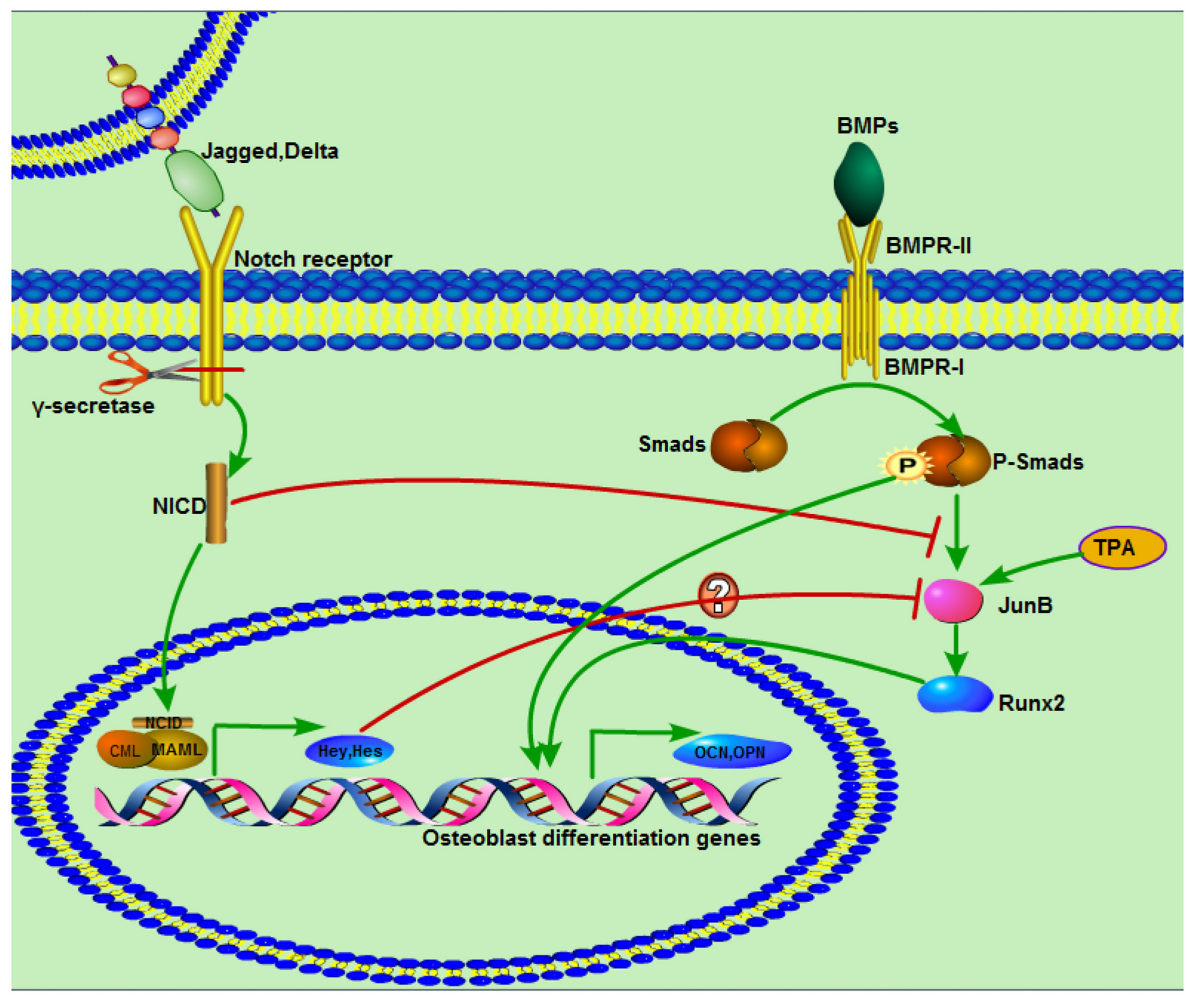
Diagram demonstrating a proposed mechanism of activated Notch signaling in C3H10T1/2 cells and interaction with BMP/Smad signaling BMP and Notch pathways are respectively activated by ligands. BMPR- I activated Smads, including Smad1, Smad5, and Smad8, are phosphorylated and then form a complex with a common Smad, Smad4, and translocate to the nucleus to regulate target gene expression through interaction with other cofactors. Notch receptors are processed by γ-secretase, and the digested product NICD is translocated to the nucleus, where is functions as an activator of target gene transcription with other co-activators. NICD suppresses BMP9/Smad signaling by inhibiting expression of JunB, followed by impacts on other osteogenesis-related genes. TPA as a JunB activator can significantly promote JunB expression.

Previous studies have demonstrated that JunB is an essential gene for bone development and that BMP2 induction of Runx2 expression can be inhibited by c-fos (A-fos), which is a dominant inhibitor of the Jun family, but that TPA as a potent JunB activator can significantly increase expression of JunB and Runx2 [27, 28, 34]. We also found that BMP9 can significantly increase JunB expression, which was consistent with earlier findings [46]. This result suggests that JunB is downstream of BMP/Smad signaling but may act as an upstream gene of Runx2. The findings of this study reveal markedly impaired expression of JunB after treatment with the NICD plasmid, and this interesting and important result possibly illuminates the inhibitory effect of NICD on BMP9-induced osteogenic differentiation [27]. Furthermore, the negative effects of NICD on ALP staining and activity, matrix mineralization formation and expression of JunB, Runx2, OPN, and OCN were reversed by TPA. In addition, JunB expression was significantly increased compared with the NICD-treated group when the Notch pathway was blocked, which further supports the inhibitory effect of activated Notch signaling on JunB expression. We also found that BMP9 both induces MSC osteogenic differentiation and causes strong up-regulation of the Hey1 transcription factor, which has been confirmed previously [20]; some reports have shown that NICD or Hey1 can inhibit Runx2 activity by directly binding to the latter [19, 21, 47]. Thus, the negative effect of NICD on BMP9-induced osteogenic differentiation is perhaps mediated directly through NICD or Hey1 or indirectly by influencing JunB expression. In general, the effect of NCID on BMP9-induced osteogenesis differentiation of C3H10T1/2 cells can be summarized by the schematic presented in Figure 7.

To investigate whether the presence of NICD has the same effect on BMP9-induced ectopic bone formation *in vivo*, cells treated with BMP9, NICD or TPA were collected and injected subcutaneously into athymic mice, as described previously [48]. Bone mass was markedly decreased in the NICD-treated group compared with the BMP9-treated group, which is consistent with previous studies [19, 33]. In addition, the significantly reduced bone volume and trabecular number indicated that early differentiation of MSCs was compromised; in contrast, the TPA-treated group was characterized by low-turnover osteopenia. The reduced expression of Runx2, JunB, OPN, and OCN in bone mass also supports the notion that early and terminal differentiation of MSCs was blocked. Interestingly, histological staining of the retrieved samples showed that formation of bone matrix, lipid droplets and cartilage matrix was significantly impaired by NICD, which may indicate that NICD not only inhibits osteogenic differentiation but also suppresses adipogenic and chondrogenic differentiation of MSCs [19, 49, 50]. This result indicated that nonclassical BMP signaling is required for commitment of C3H10T1/2 pluripotent stem cells toward the adipocyte lineage [51, 52]. According to previous studies, the Notch pathway has a crucial function in cell cycle regulation [53, 54], and our results also demonstrate that Notch signaling has the ability to promote cell proliferation and maintain the self-renewal capacity of MSCs. This result better illustrates the negative effect of NICD on BMP9-induced osteogenic differentiation [19].

Together, these findings show that activated Notch signaling has a negative impact on the BMP9-induced osteogenesis, and we are the first to demonstrate that JunB is a key regulator of skeletogenesis, affecting bone formation through BMP/Smad and Notch signaling. The novel function of JunB as a positive regulator of bone remodeling opens up new avenues for strategies that can be used for bone loss prevention.

## MATERIALS AND METHODS

### Cell lines and chemicals

C3H10T1/2 cells were obtained from ATCC (American Type Culture Collection, Manassas, VA). Recombinant adenovirus expressing exogenous BMP9 (Ad-BMP9) was kindly provided by Dr. Tong-chuan He of the University of Chicago Medical Center. The NICD overexpression plasmid was purchased from Genechem (Genechem Co., LTD, Shanghai, China). Dulbecco’s modified Eagle’s medium (DMEM) and fetal bovine serum (FBS) were both obtained from Gibco (Grand Island, NY, USA). DAPT was purchased from Cell Signaling Technology (CST; Danvers, MA, USA), and TPA was obtained from Beyotime (Beyotime Biotechnology, Shanghai, China). Anti-OCN, anti-JunB, anti-Runx2, anti-OPN and anti-β-actin antibodies were purchased from Abcam (MA, USA). Anti-Smad1/5/8, anti-p-Smad1/5/8, anti-PCNA, and anti-Cyclin D1 antibodies were obtained from Santa Cruz Biotechnology, Inc. (CA, USA). An anti-NICD antibody was purchased from CST. Unless otherwise indicated, all chemicals were purchased from Sigma-Aldrich (Saint Louis, USA).

### Cell culture

C3H10T1/2 cells were cultured at 37°C in complete DMEM supplemented with 10% FBS, 1% penicillin, and 1% streptomycin in a 5% CO_2_ incubator under a humidified atmosphere.

### Cell transfection

C3H10T1/2 cells were plated in 100-mm dishes or 6/24-well plates. When the cells were at 60-70% confluence, they were transduced with the NICD plasmid or Ad-BMP9 using LipofectamineTM 2000 (Invitrogen, Gaithersburg, MD) or Polybrene® (Santa Cruz Biotechnology, Inc.), respectively, according to the manufacturer’s recommendations.

### Alkaline phosphatase (ALP) assays

ALP activity was assessed at days 5 and 7 according to the manufacturers’ recommendations using a modified Great Escape SEAP Chemiluminescence assay (BD Clontech, Mountain View, CA) and a histochemical staining assay with a BCIP/NBT Alkaline Phosphatase Color Development Kit (Beyotime, Haimen, China). Each assay condition was performed in triplicate, and the results were repeated in at least three independent experiments. ALP activity among samples was normalized to total cellular protein levels.

### Alizarin red staining

To assess mineralized nodule formation, C3H10T1/2 cells were seeded in 24-well plates and cultured in the presence of ascorbic acid (50 mg/ml) and glycerophosphate (10 mM) after being transduced with Ad-BMP9 or the NICD plasmid. After 24 h, the cells were treated with DAPT or TPA (100 ng/ml). On day 14 following drug treatment, bone nodule formation was assessed by staining for calcium precipitation with Alizarin red S, as described previously [29].

### Flow cytometry

C3H10T1/2 cells in logarithmic growth phase were seeded in 6-well plates; after treatment with the NICD plasmid for 48 h, the cells were digested, washed 3 times with sterile phosphate-buffered saline (PBS) and fixed overnight with 70% ethanol at 4°C. Next, the cells were incubated with PI/Triton X-100 and stained for 15 min, and the cell cycle distribution was examined by flow cytometry. The experiment was repeated three times.

### Colony-forming unit-fibroblast (CFU-F) assays

C3H10T1/2 cells were plated in 65-mm dishes and transfected with the NICD plasmid when they reached 60-70% confluence; the cells were then digested and counted. Each group of cells was subsequently cultured for 8 days at a density of 1×10^3^ cells per well in 6-well plates. At the indicated time, the cells were washed three times with PBS and fixed for 10 minutes with 4% paraformaldehyde at 37°C. The paraformaldehyde was discarded, and the cells were washed three times with PBS and stained with Giemsa dye for 10 minutes; the cells were gently rinsed with running water and air dried. The cells were examined under a microscope, and a cluster of more than 50 cells was considered a colony.

### Real-time polymerase chain reaction (RT-qPCR) analysis

Total RNA was isolated from cultured cells using TRIzol Reagent (Beyotime, Haimen, China) according to the RNA extraction protocol. cDNA was synthesized from total RNA using reverse transcriptase PCR. GAPDH was used as the endogenous control. RT-qPCR was performed as described previously [30]. The RT-qPCR primers (Table 1) were designed and synthesized by Takara (Takara Bio. Inc., China). Quantification of the relative expression levels of target genes was achieved by normalization to GAPDH levels. A touchdown cycling program for RT-qPCR was performed as described previously [31].

**Table 1:** Sequences of primers used for RT-qPCR (mouse)

Gene name	Length	Primer sequence
*R*unx2	196 bp	Forward:5’ CCAACTTCCTGTGCTCCGTG 3’
		Reverse:5’ TCGTTGAACCTGGCTACTTGG 3’
*J*unB	182 bp	Forward:5’ TACCTCCCACATGCACCACC 3’
		Reverse:5’ CGCTTTCGCTCCACTTTGAT 3’
*G*APDH	117 bp	Forward:5’ GACATCAAGAAGGTAATGAAGC 3’
		Reverse:5’ GAAGGTGGAAGAGTGGGAGTT 3’
*H*ey1	123 bp	Forward:5’ TATCGGAGTTTGGGGTTTCG 3’
		Reverse:5’ TGCGTAGTTGTTGAGATGGGAG 3’
*H*es1	174 bp	Forward: 5’ GTCTAAGCCAACTGAAAACACTG 3’
		Reverse: 5’ GGTATTTCCCCAACACGCTC 3’
*H*es5	200 bp	Forward: 5’ GATGCTCAGTCCCAAGGAG 3’
		Reverse: 5’ CGAAGGCTTTGCTGTGTTTC 3’
*N*ICD1	93 bp	Forward:5’ CCGTGGATGACCTAGGCAAGT 3’
		Reverse:5’ TGTTGGCTCCGTTCTTCAGG 3’
*O*CN	199 bp	Forward:5’ TCTGACAAAGCCTTCATGTCC 3’
		Reverse:5’ AAATAGTGATACCGTAGATGCG 3’
*O*SX	132 bp	Forward:5’ GGGAGCAGAGTGCCAAGA 3’
		Reverse:5’ TACTCCTGGCGCATAGGG 3’
*I*D1	138 bp	Forward:5’ ACGACATGAACGGCTGCT 3’
		Reverse:5’ CAGCTGCAGGTCCCTGAT 3’

### Western blotting

Cells were washed three times with cold PBS and lysed in RIPA lysis buffer (Beyotime, Haimen, China) in the presence of protease and phosphatase inhibitors. The protein concentration was measured with a *bicinchoninic acid* (BCA) protein assay kit (Beyotime, Haimen, China). Equivalent amounts of protein were separated by sodium dodecyl sulfate polyacrylamide gel electrophoresis (SDS-PAGE) and then transferred to polyvinylidene difluoride (PVDF) membranes. The PVDF membranes were blocked with 5% bovine serum albumin (BSA; Solarbio, Beijing, China) in Tris-buffered saline/Tween 20 (TBST) for 2 h at 37°C and then incubated overnight with primary antibodies at 4°C. The membranes were washed 3 times with TBST and incubated with either an anti-mouse or anti-rabbit secondary antibody (1:5000; ZhongShan-Golden Bridge, Beijing, China) for 1 h at 37°C. Specific bands were visualized usingBeyoECL Plus (Beyotime, Haimen, China), as described previously [31]. All experiments were repeated three times.

### Stem cell implantation

All animal experiments were approved by the Institutional Animal Care and Use Committee (IACUC) of Chongqing Medical University. C3H10T1/2 cells at 60%-70% confluence were transduced with AdBMP9 or the NICD plasmid and further cultured for 24 h before TPA (100 ng/ml) treatment. The cells were subcutaneously injected (8×10^6^ cells/injection) into the flank of athymic nude mice (3 animals per group, 4 to 5-week-old male mice), which were purchased from HUAFUKANG (Beijing HFK Bioscience Co., Ltd., Beijing, China). At 4 weeks after implantation, the animals were killed, and the implantation sites were retrieved for microcomputed tomography (microCT) analysis, histological evaluation and other staining analyses.

### MicroCT analysis and histological evaluation

A vivaCT 40 microCT system (Scanco Medical) was used to acquire heterotopic bone data, consisting of bone mineral density (BMD), relative bone volume (BV/TV), trabecular thickness (Tb.Th), trabecular number (Tb.N) and trabecular separation (Tb.Sp) values. Retrieved tissues were decalcified with ethylenediaminetetraacetic acid (EDTA) decalcifying reagent (Beijing Solarbio Science and Technology Co., Ltd., Beijing, China) for approximately 2 weeks and subsequently embedded in paraffin. Serial sections of the embedded specimens were stained with hematoxylin and eosin (H&E), Oil red O, Alcian blue or Masson’s trichrome according to the manufacturer’s recommendations (Beijing Solarbio Science and Technology Co., Ltd., Beijing, China). A kit obtained from ZSGB-BIO (ZSGB Biotechnology, Beijing, China) was used for immunohistochemistry, which was performed according to the instructions. Bone histomorphology was assessed using a microscope (T-DH; Nikon corporation, Tokyo, Japan).

### Statistical analysis

All data were analyzed using SPSS 18.0 statistical software (SPSS Inc., Chicago, IL, USA). Results are expressed as the mean ± SD or mean ± SEM. One-way analysis of variance (ANOVA) was used to validate comparisons among groups. Comparisons of the mean between groups were carried out using the least significant difference (LSD) *t*-test. Bilateral p < 0.05 was considered to be statistically significant.
